# Supplementary material for: The role of scaffold reshaping and disassembly in dynamin driven membrane fission
Source: eLife. 2018 Dec 18;7:e39441. doi: 10.7554/eLife.39441 (PMC6355196; doi:10.7554/eLife.39441)
Supplement: Transparent reporting form [file elife-39441-transrepform.docx]

***eLife’s* transparent reporting form**

We encourage authors to provide detailed information *within their submission* to facilitate the interpretation and replication of experiments. Authors can upload supporting documentation to indicate the use of appropriate reporting guidelines for health-related research (see [EQUATOR Network](http://www.equator-network.org/%20)), life science research (see the [BioSharing Information Resource](https://biosharing.org/)), or the [ARRIVE guidelines](http://www.plosbiology.org/article/info:doi/10.1371/journal.pbio.1000412) for reporting work involving animal research. Where applicable, authors should refer to any relevant reporting standards documents in this form.

If you have any questions, please consult our Journal Policies and/or contact us: [editorial@elifesciences.org](mailto:editorial@elifesciences.org).

**Sample-size estimation**

- You should state whether an appropriate sample size was computed when the study was being designed
- You should state the statistical method of sample size computation and any required assumptions
- If no explicit power analysis was used, you should describe how you decided what sample (replicate) size (number) to use

Please outline where this information can be found within the submission (e.g., sections or figure legends), or explain why this information doesn’t apply to your submission:

n/a

**Replicates**

- You should report how often each experiment was performed
- You should include a definition of biological versus technical replication
- The data obtained should be provided and sufficient information should be provided to indicate the number of independent biological and/or technical replicates
- If you encountered any outliers, you should describe how these were handled
- Criteria for exclusion/inclusion of data should be clearly stated
- High-throughput sequence data should be uploaded before submission, with a private link for reviewers provided (these are available from both GEO and ArrayExpress)

Please outline where this information can be found within the submission (e.g., sections or figure legends), or explain why this information doesn’t apply to your submission:

The simulation trajectories presented in Figure 2f were all run in triplicate, and the outcomes of these can be seen in Figure 2 - Supplementary Figure 1; the associated data are also available in our source date files (see below).

**Statistical reporting**

- Statistical analysis methods should be described and justified
- Raw data should be presented in figures whenever informative to do so (typically when N per group is less than 10)
- For each experiment, you should identify the statistical tests used, exact values of N, definitions of center, methods of multiple test correction, and dispersion and precision measures (e.g., mean, median, SD, SEM, confidence intervals; and, for the major substantive results, a measure of effect size (e.g., Pearson's r, Cohen's d)
- Report exact p-values wherever possible alongside the summary statistics and 95% confidence intervals. These should be reported for all key questions and not only when the p-value is less than 0.05.

Please outline where this information can be found within the submission (e.g., sections or figure legends), or explain why this information doesn’t apply to your submission:

Given the still small number of trajectories we ran, we cannot do a classical statistical error analysis on their fission outcomes.

However, at one instance we claim that a change in gyration radius following the elimination of the adhesion potential is not statistically significant. For this, we simply use the standard expression for the likelihood that two Gaussian random variables with variances $\sigma_{1}^{2}$ and $\sigma_{2}^{2}$ have a separation larger than *s*, namely $\mathrm{erfc}\left[ s/\sqrt{2(\sigma_{1}^{2}+\sigma_{21}^{2})} \right]$, which in our case gives a p-value of p=0.36.

(For large datasets, or papers with a very large number of statistical tests, you may upload a single table file with tests, Ns, etc., with reference to sections in the manuscript.)

**Group allocation**

- Indicate how samples were allocated into experimental groups (in the case of clinical studies, please specify allocation to treatment method); if randomization was used, please also state if restricted randomization was applied
- Indicate if masking was used during group allocation, data collection and/or data analysis

Please outline where this information can be found within the submission (e.g., sections or figure legends), or explain why this information doesn’t apply to your submission:

n/a

**Additional data files (“source data”)**

- We encourage you to upload relevant additional data files, such as numerical data that are represented as a graph in a figure, or as a summary table
- Where provided, these should be in the most useful format, and they can be uploaded as “Source data” files linked to a main figure or table
- Include model definition files including the full list of parameters used
- Include code used for data analysis (e.g., R, MatLab)
- Avoid stating that data files are “available upon request”

Please indicate the figures or tables for which source data files have been provided:

Several figures in our article contain the results of simulation trajectories, such as for instance the size of a constricted neck as a function of time or scaffold constriction radius. These data are available as separate ASCII text files with the columns appropriately labeled. Detailed information is contained in the starting lines of these files, which begin with the comment-character ‘#’.

Here are the available data:

Figure 2f, four trajectories of gyration radius versus scaffold radius, in the files

Fig2f-con.txt	(constriction only protocol)

Fig2f-con-rot.txt	(constriction + rotation protocol)

Fig2f-con-elo.txt	(constriction + elongation protocol)

Fig2f-con-rot-elo.txt	(constriction + rotation + elongation protocol)

Figure 2, Figure supplement 1: the 3 trajectories for each sub-panel a-d:

Fig2f-supp-con.txt	(constriction only protocol, panel c)

Fig2f-supp-con-rot.txt	(constriction + rotation protocol, panel a)

Fig2f-supp-con-elo.txt	(constriction + elongation protocol, panel d)

Fig2f-supp-con-rot-elo.txt	(constriction + rotation + elongation protocol, panel b)

Figure 3, gyration radius as a function of time step, in the file

Fig3.txt	(Rg versus time)

Figure 6, scaffold radius, pitch, and rotation angle as a function of time, in the files

Fig6-radius.txt	(radius as a function of time)

Fig6-pitch.txt	(pitch as a function of time)

Fig6-angle.txt	(angle as a function of time)
